# Adapted laboratory evolution of *Thermotoga* sp. strain RQ7 under carbon starvation

**DOI:** 10.1186/s13104-022-05982-9

**Published:** 2022-03-10

**Authors:** Jyotshana Gautam, Hui Xu, Junxi Hu, Christa Pennacchio, Anna Lipzen, Joel Martin, Zhaohui Xu

**Affiliations:** 1 Department of Biological Sciences, Bowling Green State University, Bowling Green, OH 43403 USA; 2 School of Life Sciences, Minnan Normal University, 36 Xianqianzhi Street, Zhangzhou, 363000 Fujian China; 3 Department of Energy-Joint Genome Institute, 1 Cyclotron Road, Berkeley, CA 94720 USA

**Keywords:** Adapted laboratory evolution, *Thermotoga*, Starvation adaptation, SNPs, Indels

## Abstract

**Objective:**

Adaptive laboratory evolution (ALE) is an effective approach to study the evolution behavior of bacterial cultures and to select for strains with desired metabolic features. In this study, we explored the possibility of evolving *Thermotoga* sp. strain RQ7 for cellulose-degrading abilities.

**Results:**

Wild type RQ7 strain was subject to a series of transfers over six and half years with cellulose filter paper as the main and eventually the sole carbon source. Each transfer was accompanied with the addition of 50 μg of *Caldicellulosiruptor saccharolyticus* DSM 8903 genomic DNA. A total of 331 transfers were completed. No cellulose degradation was observed with the RQ7 cultures. Thirty three (33) isolates from six time points were sampled and sequenced. Nineteen (19) of the 33 isolates were unique, and the rest were duplicated clones. None of the isolates acquired *C. saccharolyticus* DNA, but all accumulated small-scale mutations throughout their genomes. Sequence analyses revealed 35 mutations that were preserved throughout the generations and another 15 mutations emerged near the end of the study. Many of the affected genes participate in phosphate metabolism, substrate transport, stress response, sensory transduction, and gene regulation.

**Supplementary Information:**

The online version contains supplementary material available at 10.1186/s13104-022-05982-9.

## Introduction

Characterized by continuous culture transfers over a prolonged period, adaptive laboratory evolution (ALE) is a procedure of exposing microbial cultures under selective pressures for prolonged periods of time, ranging from weeks to years, either through serial passages or under chemostat conditions. ALE mimics the natural selection process and selects for mutations having the tendency to optimize metabolic activities under given conditions. It is widely employed to study microbial genome evolution in a controlled laboratory setting [1–3], to select for desired phenotypes of biotechnological importance [4–7], and to optimize nutrient utilization [7–10].

*Thermotoga* species are hyperthermophilic bacteria that can produce up to 4 mol of hydrogen gas from each mole of glucose, the theoretical maximum yield of the Embden–Meyerhof pathway [11, 12]. They are able to utilize a wide range of carbon sources, such as glucose, xylose, mannose, cellobiose, starch, rice flour etc. [13, 14]. However, they have limited ability to utilize crystalline cellulose, due to a lack of exoglucanase genes [15]. This greatly constrains their application in bioenergy production, since cellulose is abundant in nature and the preferred feedstock of a sustainable biofuel industry. To help *Thermotoga* use cellulose, cellulose-degrading genes of *Caldicellulosiruptor saccharolyticus* DSM 8903 have been cloned into *T*. sp. strain RQ2 but are found to be lost in three consecutive transfers [15]. In fact, stable expression of heterogeneous genes is a common challenge in genetic engineering attempts. As an alternative approach, in this study, we attempted to evolve *T.* sp. strain RQ7 for cellulose-degrading abilities, using cellulose filter paper as the main carbon source.

The complete genome sequence of RQ7 is available [16], making it possible to keep track of the genome changes throughout the ALE process. RQ7 is also naturally competent [17]. In order to speed up the ALE process, we supplied the RQ7 cultures with genomic DNA of *C. saccharolyticus*, which has the complete set of genes needed to degrade cellulose [18] and can disassemble a piece of cellulose filter paper in 4 days (Additional file 1: Figure S1). The hypotheses were: (1) over the time, some RQ7 transformants might have the chance to take up and integrate *C. saccharolyticus* cellulose-degrading genes into their genomes; (2) these transformants would grow faster and gradually dominate the population when cellulose was supplied as the sole carbon source; (3) when such cultures occur, the filter paper should be deformed (e.g. etched or disassembled), offering visual clues on when to stop the experiment.

## Main text

### Materials and methods

#### Growth media

Two types of media were used in this study: a rich medium called SVO [19] and a selective medium. SVO uses 5 g/L of glucose, 2 g/L of yeast extract, and 2 g/L of tryptone as the carbon and nitrogen sources. The selective medium was identical to a minimal medium we previously developed [20] except replacing the glucose with a piece of Whatman^®^ Grade 1 filter paper of a surface area of 7 cm^2^. Cysteine hydrochloride was added to both media as a reducing agent at 0.5 g/L [21].

#### Adaptive laboratory evolution

The ALE process started with a wild type RQ7 culture grown in SVO (pH 8.5) (Additional file 1: Figure S2a). One ml of such culture was added to 10 ml of fresh SVO together with 50 μg of *C. saccharolyticus* DSM 8903 genomic DNA. The mixture was incubated at 77 °C for about 4 h for natural transformation. The entire 10 ml of the transformation mixture was then added to 50 ml of fresh selective medium, which was equivalent to supplying the selective medium with SVO to a final concentration of 17%, or a 6× -diluted rich medium. This was to prevent the collapse of the culture line by supplying low levels of accessible carbon sources to early cultures (Additional file 1: Figure S2a). The culture, named as NT1, was then incubated at 77 °C for 6 days to enrich potential transformants. After the incubation, 1 ml of NT1 was used to inoculate 50  ml of SVO for overnight growth to generate a boost culture. The boost culture was then used to start the next cycle for NT2. A portion of the boost culture was also preserved in 10% glycerol (v/v) and kept at −80 °C for future use. The boosting step was to revive stressed cultures (after growing in diluted medium for about a week) to a cell density high enough for the next round of transfer. We periodically tested whether the culture was ready to wean from the boosting step and noticed it by NT115.

Starting from NT115, we made several changes to our transfer procedure to simplify the procedure and increase the chance of selecting transformants (Additional file 1: Figure S2b). First, the 3-step operation was consolidated into a single step: selective cultures were directly used to inoculate the next batch, boost cultures were only used to prepare frozen stocks, and *C. saccharolyticus* DNA was directly added into the selective medium. Second, to further increase selective pressure, the SVO concentration was reduced to 9%, which was then phased out by NT212. Last, the media pH was adjusted to pH 7.2 and the growth temperature was set at 70 °C; these changes were to accommodate the potential needs of *C. saccharolyticus* genes because this bacterium has optimal growth at pH 7.0 and 70 °C [18]. Both wild type RQ7 and evolved cultures grew normally under these conditions.

#### Mutants isolation and resequencing

Single colonies were isolated from various time points throughout the ALE procedure. For handling and plating techniques as well as genomic DNA preparation, please referred to our previous publications [16, 22]. For resequencing, genomic DNA was randomly sheared into ~ 500 bp fragments, and the resulting fragments were used to create an Illumina library. This library was sequenced on Illumina NovaSeq, generating 150 bp paired-end reads. Reads were aligned to the reference genomes using BWA [23], and putative single nucleotide polymorphisms (SNPs) and small indels were called using SAMtools mpileup [24]. Putative structural variants were called using a combination of BreakDancer [25] (filtered to quality 90 +), Pindel [26], and CNVNator [27]. To locate mutations, read alignments were analyzed with Integrative Genomics Viewer (IGV) version 2.6 [28]. Variant calls with heterozygous status were filtered out because those were non-specific mapping of similar reads. After that, each variant call was manually examined with IGV. False positives resulted from sequencing errors and clustered variants mapped to repetitive regions (such CRISPR regions) were removed due to low confidence.

### Results and discussion

#### ALE experiment

The experiment started in December 2011 and lasted until June 2018. It was arbitrarily suspended to give us the time to analyze the cultures and adjust our strategies accordingly. A total of 331 transfers were completed, resulting in 331 batches of evolved bacterial populations, named as NT1 – NT331 (Additional file 1: Figure S3). For batches NT1 through NT211, it was necessary to supply the selective media with small amount of the rich medium to avoid the collapse of the culture line before desired transformants/mutants could occur. By NT212, supplying SVO had been phased out (Additional file 1: Figure S3). Cells were challenged to use filter paper as the sole carbon source. At this point, visible cloudiness of growth could no longer be observed in the selective medium, indicating a cell density less than 10^7^ per ml. However, the boost cultures still resulted in normal growth, suggesting there were enough live cells in the inoculum. In a control experiment, we started with an overnight SVO culture of wild type RQ7 and consecutively transferred it in the selective medium (filter paper as the sole carbon source, no added DNA) for 10 times. Boost cultures were obtained up to the 9th transfer but not for the 10th transfer. In contrast, our evolved cultures had survived for 120 transfers (from NT212 to NT331) in the selective medium (filter paper as the sole carbon source, DNA added), which demonstrated that active growth did occur in each culture. Otherwise, the original cells would have been diluted out by the 9th transfer, leaving no cells in the inoculum to start the next cycle. However, the filter paper pieces appeared physically intact in each bottle, without any visible sign of degradation.

#### Isolation and characterization of RQ7 mutants

To investigate what genetic changes had occurred in the evolved cultures, we isolated mutants at six time points, roughly every 55 batches: NT055, NT110, NT167, NT220, NT270, and NT331. Six DNA preparations from each batch were subject to Illumina sequencing, and a total of 33 isolates were successfully sequenced (Additional file 1: Figure S3). Clean sequence reads were compared to the two reference genomes: RQ7 (RefSeq: NZ_CP007633.1) which had been sequenced by our group [16] and *C. saccharolyticus* DSM 8903 (RefSeq: NC_009437.1). After filtering out false positive variation calls and manually examining of the alignments with IGV, we confirmed 109 RQ7 genome variants among the 33 isolates. These variations included 84 SNPs and 25 indels; 10 of the variants located in intergenic regions and the rest in CDS. Based on the occurrence of these variations, 19 unique isolates were identified (Additional file 1: Figure S3). Although the sequence depth was over 200 × in most regions, all reads were mapped to the RQ7 genome, and no read could be reliably identified as having a *C. saccharolyticus* origin. These results indicated that all isolates were RQ7 mutants surviving extreme carbon starvation. No isolate acquired *C. saccharolyticus* DNA.

#### Preserved mutations

Most of the 109 verified variations did not survive into later generations and were lost in the culture line. However, 35 mutations survived to the end of the experiment and could be evolutionarily significant, which included 29 in CDS, 5 in intergenic regions, and 1 in 23S rRNA (Table 1). Mutations in 23S rRNA and the intergenic regions were SNPs and their roles were difficult to speculate without experimental data. The 29 mutations found in CDS (Table 2) could potentially contribute to survival under starvation. It is also possible that some of these mutations were results of genome drifts over the time and carried little evolution significance. There were 15 mutations emerged in NT331 isolates (Table 3); their stability remained to be examined. Analysis of the CDS mutations revealed a common theme centered on phosphate metabolism, such as ATP generation and utilization, phosphate regulation, and nucleotide metabolism (Tables 2 and 3).

**Table 1:**
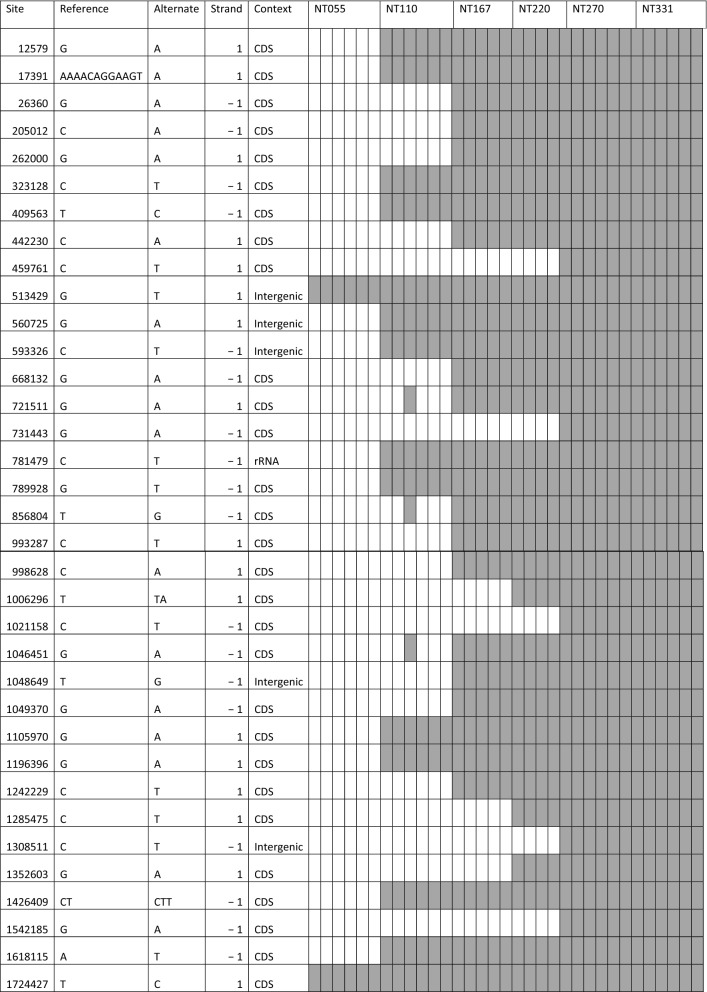
Preserved mutations

The last 33 columns represent the 33 isolates. Detected mutations are shaded

**Table 2:** Preserved mutations happened in coding sequences

Site	Locus_tag	Product and length	Base change*	Codon change
Indels				
17391	TRQ7_RS00090	Flagellar biosynthesis protein FlhB	d11: AAAACAGGAAGT → A	Frame shift, truncation
1006296	TRQ7_RS05035	Alpha-amylase	i1: T → TA	Frame shift, run-through
1426409	TRQ7_RS07315	2-hydroxyacid dehydrogenase	i1: AG → AGG	Frame shift, truncation
SNPs				
205012	TRQ7_RS01075	Methylmalonyl-CoA carboxyltransferase	Transversion: G → T	Silent: V360
262000	TRQ7_RS01400	Hypothetical protein	Transition: G → A	Silent: L346
323128	TRQ7_RS01715	Queuosine precursor transporter	Transition: G → A	Silent: T204
459761	TRQ7_RS02405	Tyrosine-tRNA ligase	Transition: C → T	Silent: V15
1724427	TRQ7_RS08830	Alpha-glucuronidase Agu4A	Transition: T → C	Silent: H107
ABC transporters				
409,563	TRQ7_RS02130	ABC transporter substrate-binding protein	Transition: A → G	Missense: Q545R
668132	TRQ7_RS03395	Sugar ABC transporter permease	Transition: C → T	Missense: A283V
1046451	TRQ7_RS05215	Sugar ABC transporter ATP-binding protein	Transition: C → T	Missense: A406V
1,049,370	TRQ7_RS05225	Sugar ABC transporter substrate-binding protein	Transition: C → T	Missense: P115S
1542185	TRQ7_RS07940	ABC transporter ATP-binding protein	Transition: C → T	Missense: P290S
Stress response				
442230	TRQ7_RS02305	PhoH family protein	Transversion: C → A	Missense: S123R
789928	TRQ7_RS04030	Sodium-translocating pyrophosphatase	Transversion: C → A	Missense: A461E
993287	TRQ7_RS04975	5'/3'-nucleotidase SurE	Transition: C → T	Missense: P50L
1105970	TRQ7_RS05480	Ribose-phosphate pyrophosphokinase	Transition: G → A	Missense: A83T
1196396	TRQ7_RS05995	Phosphate signaling complex protein PhoU	Transition: G → A	Missense: G83S
Sensing and regulation				
856804	TRQ7_RS04355	ROK family transcriptional regulator	Transversion: A → C	Missense: N12T
998628	TRQ7_RS05010	Response regulator transcription factor	Transversion: C → A	Missense: L189M
1242229	TRQ7_RS06235	Transcriptional repressor	Transition: C → T	Nonsense: Q9
1285475	TRQ7_RS06565	RNA polymerase sigma factor RpoD	Transition: C → T	Missense: L280F
1618115	TRQ7_RS08290	Sensor domain-containing diguanylate cyclase	Transversion: T → A	Missense: V390E
Others				
12579	TRQ7_RS00060	Ribonuclease HII	Transition: G → A	Missense: A237T
26360	TRQ7_RS00155	Galactose-1-phosphate uridylyltransferase	Transition: C → T	Missense: P274L
721511	TRQ7_RS03655	UDP-N-acetylmuramoyl-tripeptide–D-alanyl-D- alanine ligase	Transition: G → A	Missense: D148N
731443	TRQ7_RS03700	NADH-quinone oxidoreductase subunit NuoE	Transition: C → T	Missense: S15L
1021158	TRQ7_RS05125	Hypothetical protein	Transition: G → A	Missense: G148D
1352603	TRQ7_RS06890	Hypothetical protein	Transition: G → A	Missense: V418I

^*^d11: deletion of 11 bases; i1: insertion of 1 base

**Table 3:**
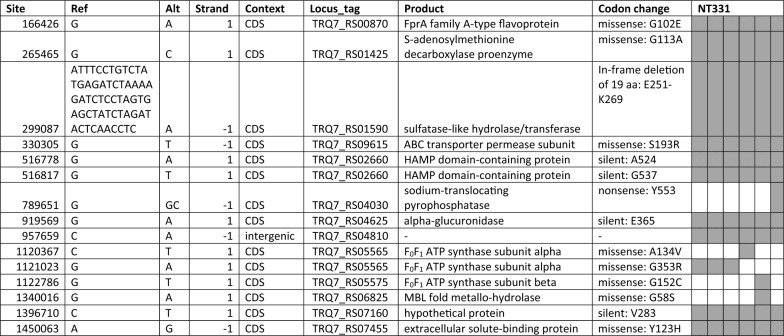
Mutations emerged in NT331

The last six columns represent the six NT331 isolates. Detected mutations are shaded

## Conclusions

*T.* sp. strain RQ7 survived 331 ALE transfers under carbon starvation. Their genomes accumulated dozens of small-scale mutations but no integration of *C. saccharolyticus* DNA. They did not evolve the desired trait to utilize cellulose. Since cells could only survive beyond 10 transfers when *C. saccharolyticus* DNA was supplied, we believe that under these extreme starvation conditions cells were utilizing the added DNA as the main carbon source to support growth. This is rather encouraging, because using environmental DNA as a nutrient source is a perceived role of natural transformation, and many species only become naturally competent when they are starving [29].

## Limitations of the study

Natural transformation are rare events and are largely subject to chances. Transforming a RQ7 cell to a cellulose-degrading strain would require the acquisition of many genes and numerous natural transformation events. Six and half years of ALE is too short to allow the wild type RQ7 strain to pick up foreign genes and evolve desired traits. Longer periods of experiments are necessary. Higher concentrations of donor DNA might also help.

## Supplementary Information

**Additional file 1: Figure S1**. Degradation of cellulose filter paper by *C. saccharolyticus* DSM 8903. **Figure S2**. ALE procedures. **Figure S3**. Timeline of the major events of ALE and the sampling points of mutants.

## Data Availability

The sequencing data in this study are available in NCBI BioProject with accession numbers PRJNA568833-PRJNA568851, PRJNA568854-PRJNA568862, PRJNA582349, PRJNA584080-PRJNA584083.

## Abbreviations

ALE: Adapted laboratory evolution
CDS: Coding sequence
Indel: Insertion or deletion
IGV: Integrative Genomics Viewer
SNP: Single nucleotide polymorphism

## References

[1] Woods RJ, Barrick JE, Cooper TF, Shrestha U, Kauth MR, Lenski RE (2011). Second-order selection for evolvability in a large *Escherichia coli* population. Science.

[2] Sniegowski PD, Gerrish PJ, Lenski RE (1997). Evolution of high mutation rates in experimental populations of *E coli*. Nature.

[3] Singh R, Gradnigo J, White D, Lipzen A, Martin J, Schackwitz W, Moriyama E, Blum P (2015). Complete genome sequence of an evolved *thermotoga maritima* isolate. Genome Announc.

[4] Dragosits M, Mattanovich D (2013). Adaptive laboratory evolution—principles and applications for biotechnology. Microb Cell Fact.

[5] Ai C, McCarthy S, Eckrich V, Rudrappa D, Qiu G, Blum P (2016). Increased acid resistance of the archaeon, Metallosphaera sedula by adaptive laboratory evolution. J Ind Microbiol Biotechnol.

[6] Jilani SB, Venigalla SSK, Mattam AJ, Dev C, Yazdani SS (2017). Improvement in ethanol productivity of engineered *E coli* strain SSY13 in defined medium via adaptive evolution. J Ind Microbiol Biotechnol.

[7] Kim NY, Kim SN, Kim OB (2018). Long-term adaptation of *Escherichia coli* to methanogenic co-culture enhanced succinate production from crude glycerol. J Ind Microbiol Biotechnol.

[8] Weikert C, Sauer U, Bailey JE (1997). Use of a glycerol-limited, long-term chemostat for isolation of *Escherichia coli* mutants with improved physiological properties. Microbiology.

[9] Summers ZM, Ueki T, Ismail W, Haveman SA, Lovley DR (2012). Laboratory evolution of Geobacter sulfurreducens for enhanced growth on lactate via a single-base-pair substitution in a transcriptional regulator. ISME J.

[10] Shen Y, Chen X, Peng B, Chen L, Hou J, Bao X (2012). An efficient xylose-fermenting recombinant *Saccharomyces cerevisiae* strain obtained through adaptive evolution and its global transcription profile. Appl Microbiol Biotechnol.

[11] Schröder C, Selig M, Schönheit P (1994). Glucose fermentation to acetate, CO_2_ and H_2_ in the anaerobic hyperthermophilic eubacterium *Thermotoga maritima*: involvement of the Embden–Meyerhof pathway. Arch Microbiol.

[12] Huber R, Langworthy TA, König H, Thomm M, Woese CR, Sleytr UB, Stetter KO (1986). *Thermotoga maritima* sp. Nov. represents a new genus of unique extremely thermophilic eubacteria growing up to 90°C. Arch Microbiol.

[13] Yu X, Drapcho CM (2011). Hydrogen production by the hyperthermophilic bacterium *Thermotoga neapolitana* using agricultural-based carbon and nitrogen sources. Biol Eng Trans.

[14] Chhabra SR, Shockley KR, Conners SB, Scott KL, Wolfinger RD, Kelly RM (2003). Carbohydrate-induced differential gene expression patterns in the hyperthermophilic bacterium *Thermotoga maritima*. J Biol Chem.

[15] Xu H, Han D, Xu Z (2015). Expression of Heterologous Cellulases in Thermotoga sp. Strain RQ2. Biomed Res Int.

[16] Xu Z, Puranik R, Hu J, Xu H, Han D (2017). Complete genome sequence of Thermotoga sp. strain RQ7. Stand Genomic Sci.

[17] Han D, Xu H, Puranik R, Xu Z (2014). Natural transformation of Thermotoga sp. strain RQ7. BMC Biotechnol.

[18] Rainey FA, Donnison AM, Janssen PH, Saul D, Rodrigo A, Bergquist PL, Daniel RM, Stackebrandt E, Morgan HW (1994). Description of Caldicellulosiruptor saccharolyticus gen. nov., sp. nov: an obligately anaerobic, extremely thermophilic, cellulolytic bacterium. FEMS Microbiol Lett.

[19] Van Ooteghem SA, Beer SK, Yue PC (2002). Hydrogen production by the thermophilic bacterium *Thermotoga neapolitana*. Appl Biochem Biotechnol.

[20] Han D, Xu Z (2017). Development of a pyrE-based selective system for Thermotoga sp. strain RQ7. Extremophiles.

[21] Uchino Y, Ken-Ichiro S (2011). A simple preparation of liquid media for the cultivation of strict anaerobes. J Pet Environ Biotechnol.

[22] Han D, Norris SM, Xu Z (2012). Construction and transformation of a Thermotoga-*E. coli* shuttle vector. BMC Biotechnol.

[23] Li H, Durbin R (2010). Fast and accurate long-read alignment with Burrows–Wheeler transform. Bioinformatics.

[24] Li H, Handsaker B, Wysoker A, Fennell T, Ruan J, Homer N, Marth G, Abecasis G, Durbin R (2009). 1000 genome project data processing subgroup: the sequence alignment/map format and SAMtools. Bioinformatics.

[25] Chen K, Wallis JW, McLellan MD, Larson DE, Kalicki JM, Pohl CS, McGrath SD, Wendl MC, Zhang Q, Locke DP, Shi X, Fulton RS, Ley TJ, Wilson RK, Ding L, Mardis ER (2009). BreakDancer: an algorithm for high-resolution mapping of genomic structural variation. Nat Methods.

[26] Ye K, Schulz MH, Long Q, Apweiler R, Ning Z (2009). Pindel: a pattern growth approach to detect break points of large deletions and medium sized insertions from paired-end short reads. Bioinformatics.

[27] Abyzov A, Urban AE, Snyder M, Gerstein M (2011). CNVnator: an approach to discover, genotype, and characterize typical and atypical CNVs from family and population genome sequencing. Genome Res.

[28] Robinson JT, Thorvaldsdóttir H, Wenger AM, Zehir A, Mesirov JP (2017). Variant review with the integrative genomics viewer. Cancer Res.

[29] Redfield RJ (1993). Genes for breakfast: The Have-Your-Cake and-Eat-Lt-Too of bacterial transformation. J Hered.

