# Supplementary material for: Adapted laboratory evolution of Thermotoga sp. strain RQ7 under carbon starvation
Source: BMC Res Notes. 2022 Mar 10;15:99. doi: 10.1186/s13104-022-05982-9 (PMC8908640; doi:10.1186/s13104-022-05982-9)

**Figure S1.** Degradation of cellulose filter paper by *C. saccharolyticus* DSM 8903. Top: Negative control; filter paper was incubated at 70°C for 18 days in a modified ATCC medium 1368 where cellobiose was omitted; the filter paper remained intact. Bottom: Same medium inoculated with *C. saccharolyticus* and incubated at 70°C for 4 days; the filter paper was completely dismantled and became fine, freely floating fibers.


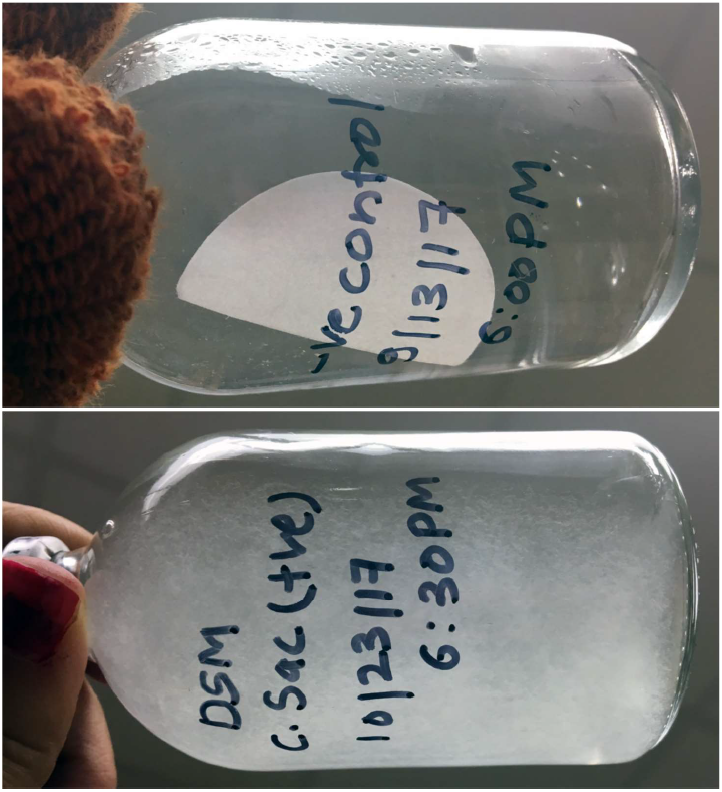


**Figure S2.** ALE procedures. (a) Three-step transfers for NT1-115 and (b) one-step transfers for NT116-331. SVO medium is shown in light yellow, and selective medium contains a piece of filter paper in the shape of a half circle. Half-filled bottles contained 50 ml of growth media, the less-filled contained 20 ml, and the least-filled had 10 ml.


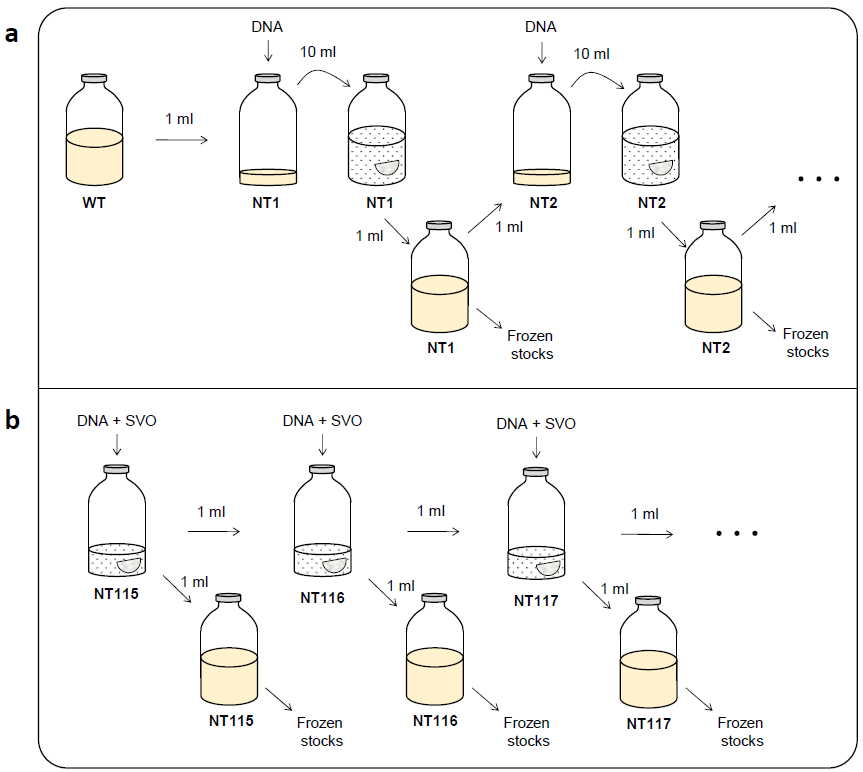


**Figure S3.** Timeline of the major events of ALE (top) and the sampling points of mutants (bottom). The serial transfers started as a 3-step procedure but was simplified to 1-step by the time of NT115. Medium pH was also changed from pH 8.5 to 7.2 at that time. In the beginning, the selective medium contained 17% of SVO (v/v). The level of SVO was phased out by NT212. Strains in the boxes had their genomes successfully sequenced. Identical isolates of the same batch are underscored.


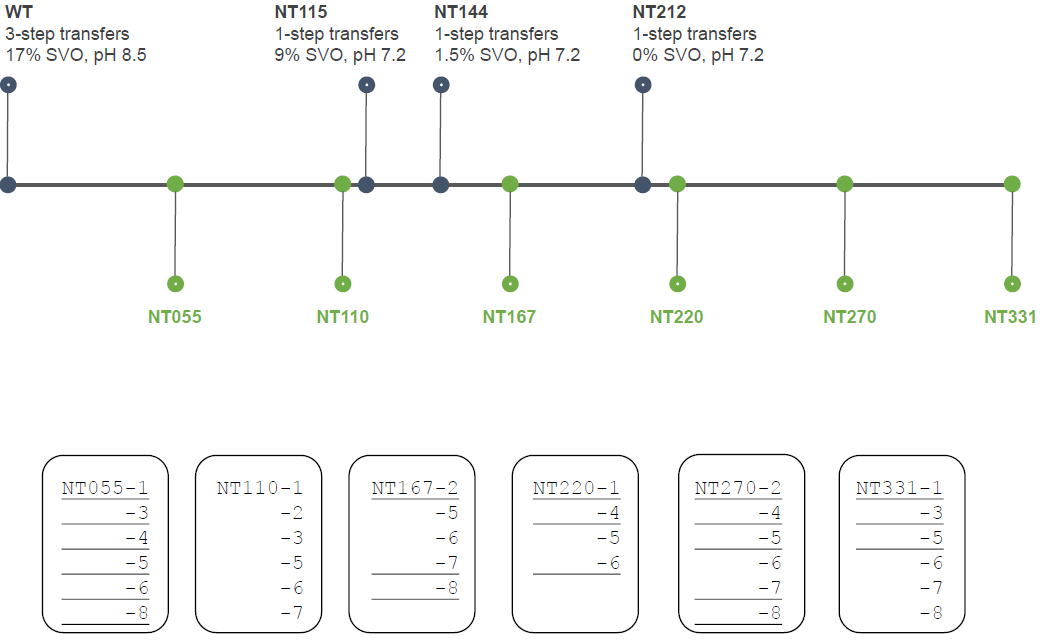

Supplement: Supplementary file 1 — Additional file 1: Figure S1. Degradation of cellulose filter paper by C. saccharolyticus DSM 8903. Figure S2. ALE procedures. Figure S3. Timeline of the major events of ALE and the sampling points of mutants. [file 13104_2022_5982_MOESM1_ESM.docx]
